# Hierarchically Assembled Nanofiber Scaffolds with Dual Growth Factor Gradients Promote Skin Wound Healing Through Rapid Cell Recruitment

**DOI:** 10.1002/advs.202309993

**Published:** 2024-02-07

**Authors:** Ruyi Fan, Chuwei Zhang, Fei Li, Bo Li, Alec McCarthy, Yi Zhang, Shixuan Chen, Lin Zhang

**Affiliations:** ^1^ Department of Histology and Embryology School of Basic Medical Sciences Southern Medical University Guangzhou 510515 China; ^2^ National Medical Products Administration (NMPA) and Guangdong Medical Products Administration (GDMPA) Key Laboratory for Safety Evaluation of Cosmetics Guangzhou 510515 China; ^3^ Zhejiang Engineering Research Center for Tissue Repair Materials Wenzhou Institute University of Chinese Academy of Sciences Wenzhou Zhejiang 325000 China; ^4^ Department of Burn and Plastic Surgery Affiliated Hospital of Nantong University Nantong 226001 China; ^5^ Department of Surgery – Transplant Holland Regenerative Medicine Program University of Nebraska Medical Center Omaha Nebraska 68105 USA

**Keywords:** cell recruitment, dual gradients, growth factors, radially aligned nanofiber scaffold, skin wound healing

## Abstract

To address current challenges in effectively treating large skin defects caused by trauma in clinical medicine, the fabrication, and evaluation of a novel radially aligned nanofiber scaffold (RAS) with dual growth factor gradients is presented. These aligned nanofibers and the scaffold's spatial design provide many all‐around “highways” for cell migration from the edge of the wound to the center area. Besides, the chemotaxis induced by two growth factor gradients further promotes cell migration. Incorporating epidermal growth factor (EGF) aids in the proliferation and differentiation of basal layer cells in the epidermis, augmenting the scaffold's ability to promote epidermal regeneration. Concurrently, the scaffold‐bound vascular endothelial growth factor (VEGF) recruits vascular endothelial cells at the wound's center, resulting in angiogenesis and improving blood supply and nutrient delivery, which is critical for granulation tissue regeneration. The RAS+EGF+VEGF group demonstrates superior performance in wound immune regulation, wound closure, hair follicle regeneration, and ECM deposition and remodeling compared to other groups. This study highlights the promising potential of hierarchically assembled nanofiber scaffolds with dual growth factor gradients for wound repair and tissue regeneration applications.

## Graphical abstract

The fabrication and evaluation of a novel radially aligned nanofiber scaffold with dual growth factor gradients are presented. These aligned nanofibers and the scaffold's spatial design provide many all‐around “highways” for cell migration from the edge of the wound to the center area. Besides, the chemotaxis induced by two growth factor gradients further promotes cell migration.

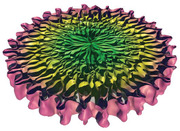

## 1 Introduction

The prevalence of various acute and chronic skin wounds, including injuries from accidents, burns, electrical and chemical exposures, diabetic foot ulcers, and pressure sores, persist as challenges to dermatologic surgery.^[^ ^1^ ^]^ The current clinic's wound management primarily focuses on debridement and infection prevention.^[^ ^2^ ^]^ Then, negative pressure treatment is performed to improve blood circulation to the wound site to accelerate granulation tissue formation.^[^ ^3^ ^]^ It inspires us that debridement can provide a favorable wound bed for wound repair, and the negative pressure treatment can enhance the repair activity of the wound bed. Therefore, the key point is still how to promote granulation tissue formation.

Various biomaterial scaffolds with distinct physical and chemical properties have been developed to aid wound repair.^[^ ^4^ ^]^ These include 3D‐printed scaffolds,^[^ ^5^ ^]^ hydrogels,^[^ ^6^ ^]^ electrospun or fiber‐based scaffolds,^[^ ^7^ ^]^ and freeze‐dried scaffolds,^[^ ^8^ ^]^ all of which offer beneficial features such as high porosity, ample surface area, moisture retention, exudate absorption, appropriate mechanical strength, and biodegradability.^[^ ^9^ ^]^ Despite these advances, there remains room for further optimization, particularly in enhancing the scaffold's structural similarities with endogenous extracellular matrix (ECM) nanostructures. Nanostructured scaffolds are crucial for directing cell behavior during tissue regeneration, including adhesion, migration, proliferation, and ECM production. They also play a role in stem cell modulation and the preservation of pluripotency.^[^ ^10^ ^]^ Herein, we engineered a novel class of 3D hierarchically assembled nanofiber scaffolds, which featured predesigned nanotopographic cues achieved through electrospinning and modified gas‐foaming expansion.^[^ ^11^ ^]^ Among these 3D nanofiber scaffolds, a 3D radially aligned scaffold (RAS) exhibits a radially distributed channel structure that has shown considerable promise in directing cell migration,^[^ ^11b^ ^]^ which has great potential for wound healing application.

Growth factors play an essential role during tissue regeneration.^[^ ^12^ ^]^ A gradient distribution of growth factors is shown in the human body from secretory cells to the local area. Compositional gradients play a critical role in developmental biology, tissue homeostasis, and tissue repair by enabling cells to infer their spatial location and determine their fate accordingly.^[^ ^13^ ^]^ During wound healing, epidermal growth factor (EGF) plays a critical role in epidermal regeneration by selectively binding to receptors, particularly in the basal layer of the epidermis, thus stimulating epidermal cell proliferation and aiding in tissue reconstruction.^[^ ^14^ ^]^ Vascular endothelial growth factor (VEGF), known for its potent angiogenic properties, fosters the proliferation and differentiation of vascular endothelial cells and increases their permeability. Therefore, strategically incorporating these growth factors in gradient form is designed to provide spatial cues that optimize cellular behaviors such as migration, adhesion, and chemotaxis.

Taken together, we hypothesize that the spatial structure of RAS in combination with two growth factor gradients could promote wound healing‐related cells, including keratinocytes, fibroblasts, endothelial cells, and immune cells, to migrate from surrounding healthy tissue to the wound area, then enhancing granulation tissue formation, re‐epithelialization, and tissue remodeling. In the presented study, we assess the scaffold's improved capacity for cellular recruitment, its effectiveness in promoting epidermal regeneration and angiogenesis, and its potential to reduce scarring in wound healing applications.

## 2 Result

### 2.1 The Preparation and Characterization of RAS

**Figure 1:**
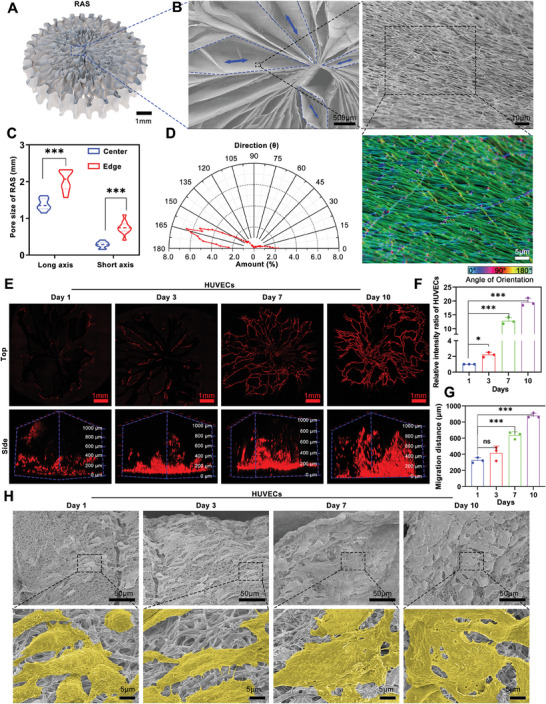
The preparation and characterization of 3D radially aligned scaffold (RAS). A,B) The RAS exhibits a radial long‐channel structure, and each channel consists of aligned PCL nanofiber. C) The quantification of pore size within the RAS. D) The characterization of fiber alignment within the RAS. E,F) The proliferation of HUVECs seeded on the RAS for 1, 3, 7, and 10 days respectively. G) The migration distance of HUVECs from the bottom to the top site of RAS at each indicated time point. (H) SEM images visualize the proliferation of HUVECs on the surface of RAS. ^*^ *p* < 0.05, ^***^ *p* < 0.001.

A 2D oriented nanofiber film was transformed into an expanded 3D radially aligned scaffold (RAS) using the previously established modified gas‐foaming method (**Figure** **1**A). A top view of the RAS revealed a stratified nanofiber film with radially aligned nanofibers and radially distributed channels (Figure 1B). The average lengths of the long and short axes of the channels in the central region of the RAS were 1.381 ± 0.1555 and 0.2842 ± 0.0804 mm, respectively. In the outer region, these measurements were 2.037 ± 0.2816 mm for the long axis and 0.7439 ± 0.1824 mm for the short axis (Figure 1C). The directional alignment of nanofibers was quantified using the ImageJ plugin OrientationJ, which showed a predominantly uniform orientation highlighted by a green pseudo‐color (Figure 1D).

The biocompatibility of the RAS was then evaluated. The distribution of human umbilical vein endothelial cells (HUVECs) on the scaffold surface was uniform postinoculation, with significant proliferation noted at 1, 3, 7, and 10 days of culture (Figure 1E). Confocal microscopy revealed a substantial increase in the fluorescence intensity of HUVECs, suggesting active cell proliferation (Figure 1F). Lateral confocal imaging demonstrated rapid cell migration within the scaffold, with distances covered by migrating cells progressively increasing to 326.2 ± 32.93 micrometers, 416.0 ± 86.4 micrometers, and 635.9 ± 43.15 micrometers after 1, 3, and 7 and eventually reaching a length of 878.4 ± 31.88 micrometers by day 10 (Figure 1G). SEM images captured the cells adhering to the nanofiber scaffold surface on day 1, forming distinct cytopseudopods by day 3. By days 7 and 10, fusion events between proliferating cells were evident (Figure 1H).

### 2.2 The Preparation and Functional Identification of RAS with Dual Drug Gradients

**Figure 2:**
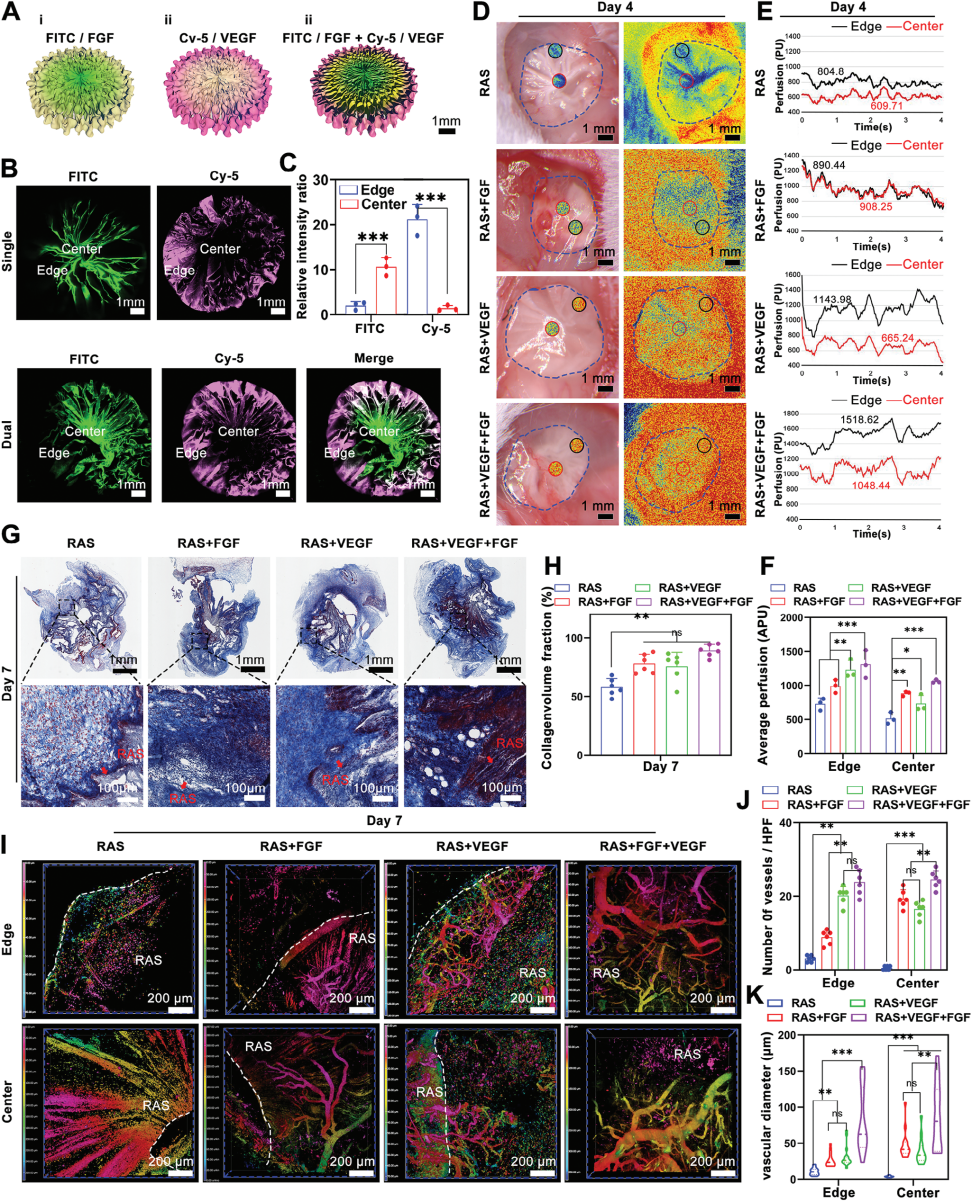
The preparation and functional identification of RAS with dual drug gradients. A) The schematic illustrates one drug gradient from the center to the periphery (FITC) or from the periphery to the center (Cy‐5), and two drug gradients, one drug from the center to the periphery (FITC) and another one from the periphery to the center (Cy‐5). B) The preparation of RAS with one drug gradient and two drug gradients. C) The quantification of relative fluorescence intensity of FTTC and Cy‐5 from center to edge in the RAS with dual gradients. D) Laser speck images of the RAS, RAS+FGF, RAS+VEGF, and RAS+VEGF+FGF groups show blood perfusion in the scaffold and surrounding tissue areas after 7 days of treatment. E) The blood perfusion in the center and edge area of RAS, RAS+FGF, RAS+VEGF, and RAS+VEGF+FGF groups after 7 days of treatment. F) The average perfusion in the center and edge area of RAS, RAS+FGF, RAS+VEGF, and RAS+VEGF+FGF groups after 7 days of treatment. G) The trichrome staining shows collagen deposition in the RAS, RAS+FGF, RAS+VEGF, and RAS+VEGF+FGF groups after 7 days of treatment. I) Tissue‐clearing technology visualizes the blood vessels in the interface between RAS and surrounding tissues after 7 days of treatment. J) The average numbers of blood vessels in the interface area of RAS, RAS+FGF, RAS+VEGF, and RAS+VEGF+FGF groups after 7 days of treatment. K) The average blood vessel diameter of RAS, RAS+FGF, RAS+VEGF, and RAS+VEGF+FGF groups after 7 days of treatment. ^*^ *p* < 0.05, ^**^ *p* < 0.01, ^***^ *p* < 0.001.

Fluorescent substances were used to simulate drugs and demonstrate the formation of bidirectional composition gradients in the RAS. As shown in **Figure** **2**A, a FITC solution was applied to the central region of the RAS, while a Cy‐5 solution was introduced to the periphery, allowing both to diffuse. The resulting fluorescence images showed a radial FITC content gradient with a more vigorous intensity in the center than in the surrounding areas. Conversely, the Cy‐5 content decreased from the periphery toward the center (Figure 2B,C).

In vivo, subcutaneous implantation confirmed the formation of growth factor gradients. Different RAS types were implanted: a blank RAS group, RAS with a graded FGF gradient from center to edge (RAS+FGF), RAS with a graded VEGF gradient from edge to center (RAS+VEGF), and RAS with dual growth factor gradients (RAS+VEGF+FGF). After 1 week of implantation, the blood flow in the tissue capsule was measured using a Laser speck imaging system over 4 s, producing a pseudo‐color map where warmer colors indicated higher perfusion (Figure 2D–F). The distribution of blood vessels correlated with the scaffold's radial structure. Larger apertures and channels in the scaffold promoted vascular growth toward the center. The RAS group showed partial perfusion in the periphery with a relative volume of 804.8 within 4 s, while the center had a lower volume of 609.71. The peripheral areas of the RAS+VEGF and RAS+VEGF+FGF groups showed higher perfusion volumes, with 4‐s relative volumes of 1143.98 and 1518.62, respectively, which was significantly higher than in the RAS and RAS+FGF groups (Figure 2E,F). Suggesting the gradient VEGF loaded at the edge of the RAS can effectively promote the growth of blood vessels into the target area. Masson trichrome staining indicated more collagen in the center of the scaffold for all growth factor‐loaded groups compared to the RAS group (Figure 2G,H). Statistical analysis showed significant differences between the effects of VEGF and FGF gradients on collagen production (Figure 2F,H).

The tissue‐clearing technique further visualized the ingrowth of blood vessels (Figure 2I). Confocal imaging showed sporadic neovascularization with small vessels in the RAS group. The RAS+FGF group had limited neovascularization, while the RAS+VEGF and RAS+VEGF+FGF groups displayed a dense vascular network with larger new vessels at the scaffold's edge. The mean vessel diameters at the scaffold margin were 10.85 ± 4.91 micrometers for the RAS group, 26.25 ± 9.530 micrometers for the RAS+FGF group, 31.47 ± 14.9 micrometers for the RAS+VEGF group, and 82.03 ± 44.07 micrometers for the RAS+VEGF+FGF group. No new vessels formed in the central region of the RAS group, while the other three groups showed significant vascular networks, especially the RAS+VEGF+FGF group. The average vascular diameters in the central region were 3.706 ± 1.140 micrometers for the RAS group, 47.39 ± 19.38 micrometers for the RAS+FGF group, 37.97 ± 17.83 micrometers for the RAS+VEGF group, and 86.49 ± 45.66 micrometers for the RAS+VEGF+FGF group (Figure 2J,K). The CD31 immunohistochemical staining also showed higher neovascularization density in the surrounding areas of the RAS+FGF and RAS+VEGF+FGF groups (Figure S1A,B, Supporting Information).

### 2.3 In Vivo Wound Healing Assessment and Histological Observations

**Figure 3:**
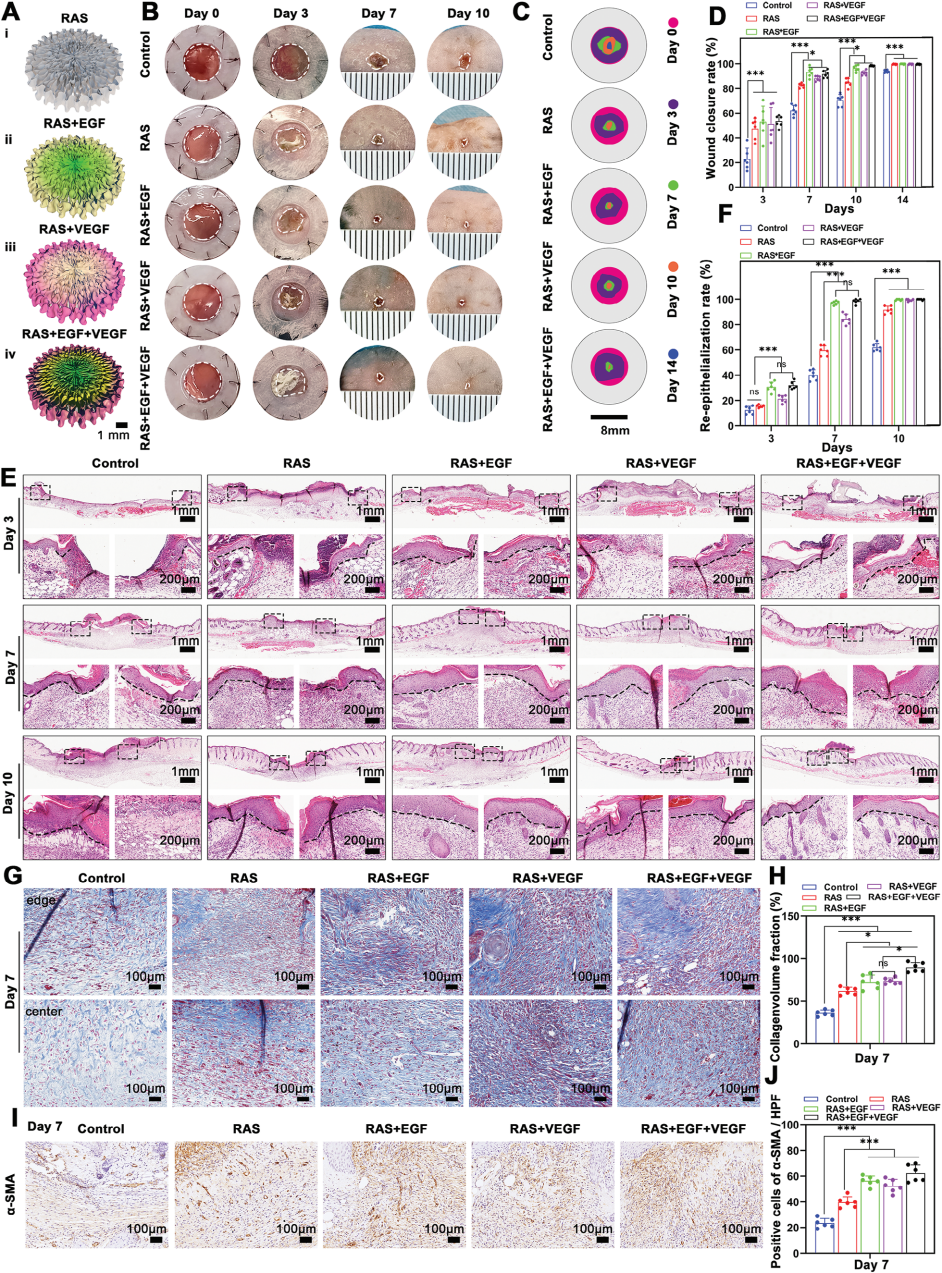
In vivo wound healing assessment and histological observations. A) Schematics illustrate that acute skin wounds were treated with RAS, RAS+EGF, RAS+VEGF, and RAS+EGF+VEGF, respectively, and the wounds were treated without any treatment as a control. B) Photographs of skin wounds of Control, RAS, RAS+EGF, RAS+VEGF, and RAS+EGF+VEGF groups at each indicated time point. C,D) The quantification of the wound closure rate of each group at 3, 7, 10, and 14 days. E) The histological observations in the wound area of the Control, RAS, RAS+EGF, RAS+VEGF, and RAS+EGF+VEGF groups at days 3, 7, and 10. F) The re‐epithelialization rate of wounds treated with RAS, RAS+EGF, RAS+VEGF, and RAS+EGF+VEGF for 3, 7, and 10 days. G) The trichrome staining shows collagen deposition in the wound center area of the Control, RAS, RAS+EGF, RAS+VEGF, and RAS+EGF+VEGF groups after 7 days of treatment. H) The quantification of newly deposited collagen fibers in the wound area of the Control, RAS, RAS+EGF, RAS+VEGF, and RAS+EGF+VEGF groups after 7 days of treatment. I,J) Immunohistochemical staining and quantification of α‐SMA in wound center area of Control, RAS, RAS+EGF, RAS+VEGF, and RAS+EGF+VEGF groups after 7 days of treatment. ^*^ *p* < 0.05, ^***^ *p* < 0.001.

The healing efficacy of the RAS with dual growth factor gradients was further validated using a murine skin wound model. Gradients of EGF and VEGF were established within and around the scaffolds to enhance keratinocyte migration at the wound margin and promote angiogenesis at the wound center, respectively (**Figure** **3**A). The RAS group, RAS+EGF group, RAS+VEGF group, and RAS+EGF+VEGF group all exhibited accelerated wound closure at each indicated time point compared to the Control group, with the RAS+EGF group and the RAS+EGF+VEGF group showing particularly significant effects. After 14 days of treatment, the RAS+EGF+VEGF group had the most minor residual wounds (Figure 3B–D).

Histological analysis was performed to assess the re‐epithelialization, granulation tissue formation, and ECM deposition. HE staining indicated that after 7 days, re‐epithelialization in the Control group was significantly lower (40.17 ± 4.167%) compared to the experimental groups. The RAS+EGF and RAS+EGF+VEGF groups showed near‐complete re‐epithelialization (97.17 ± 1.472% and 98.50 ± 1.871%, respectively). All experimental groups exhibited notable granulation tissue formation, with regenerated hair follicles appearing in the wound's central area after 10 days in the RAS+EGF+VEGF group, indicating enhanced healing quality (Figure 3E,F). Moreover, trichrome staining for collagen deposition showed significant collagen presence in the wound center and periphery after 7 days of treatment in the RAS+VEGF and RAS+EGF+VEGF groups (Figure 3G,H). Finally, Higher α‐SMA expression levels were observed in the experimental groups compared to the Control group after 7 days of treatment. In addition, the expression of α‐SMA in the RAS+EGF, RAS+VEGF, and RAS+EGF+VEGF groups showed even higher levels than the RAS group alone (Figure 3I,J).

### 2.4 RAS Promotes Re‐Epithelialization of Skin Wounds

**Figure 4:**
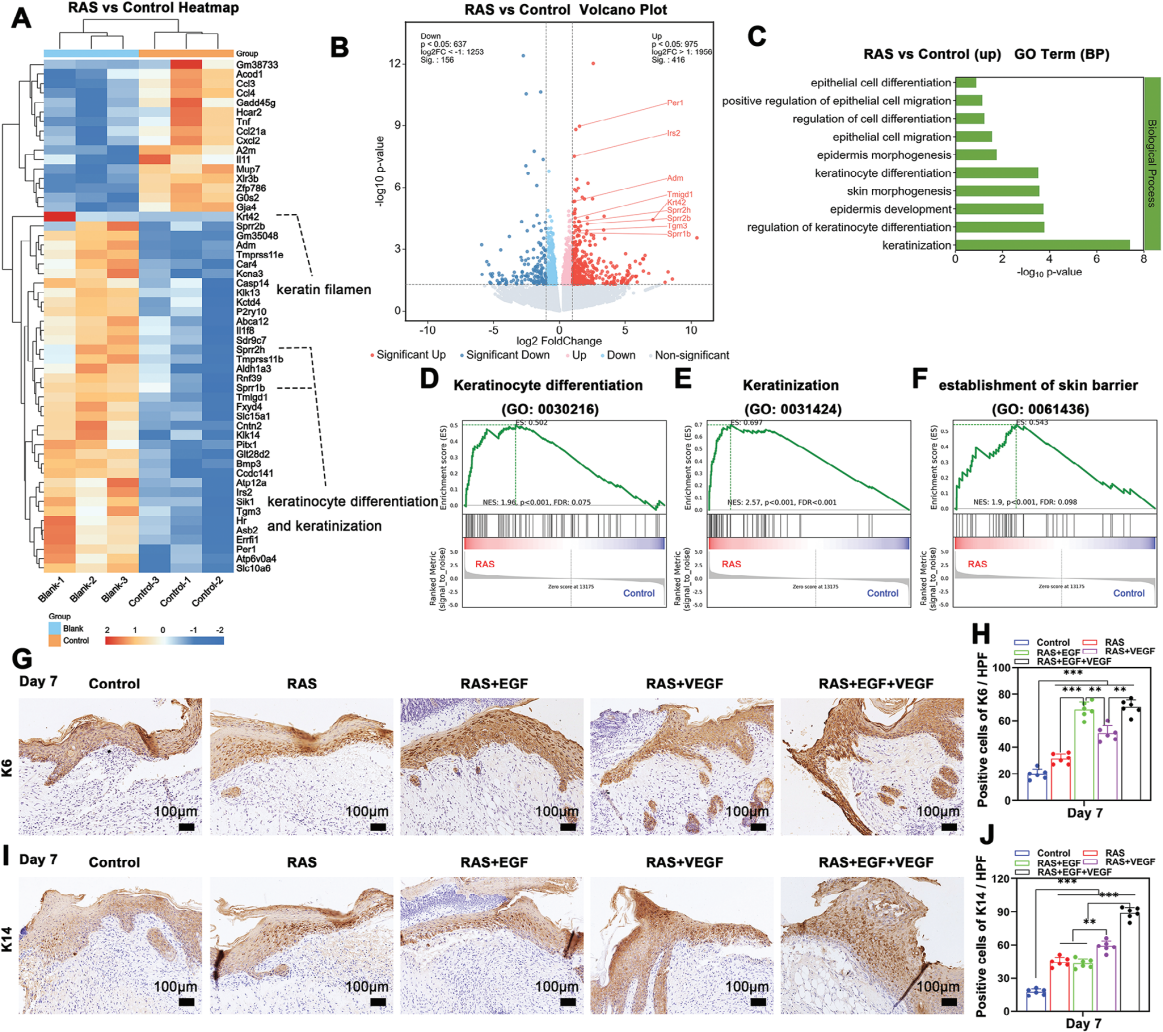
RAS promotes re‐epithelialization of skin wounds. A,B) The heatmap and volcano plot illustrate the differentially expressed genes related to keratinocytes’ activities of the RAS group compared to the control group. C) The GO function enrichment analysis of these differentially expressed genes. D–F) The gene set enrichment analysis (GSEA) of these differentially expressed genes. G,H) Immunohistochemical staining and quantification of K6 in wound center area of Control, RAS, RAS+EGF, RAS+VEGF, and RAS+EGF+VEGF groups after 7 days of treatment. I,J) Immunohistochemical staining and quantification of K14 in wound center area of Control, RAS, RAS+EGF, RAS+VEGF, and RAS+EGF+VEGF groups after 7 days of treatment. ^**^ *p* < 0.01, ^***^ *p* < 0.001.

On the 7th day postinjury, RNA‐seq was utilized to analyze the activity of various cells involved in wound repair. The heatmap and Volcano Plot analysis identified 572 differentially expressed genes (DEGs) between the RAS and Control groups. Genes significantly upregulated in the RAS group were primarily associated with keratinocyte differentiation, keratinization, and keratin filament formation (**Figure** **4**A,B). GO enrichment analysis highlighted that biological processes such as epidermal keratinization, keratinocyte differentiation, epidermal development, and morphogenesis were all upregulated in the RAS group compared to the Control group (Figure 4C). This suggests that RAS notably promotes epidermal regeneration. GSEA analysis further confirmed the RAS group's enhanced performance in keratinocyte differentiation, keratinization, and skin barrier establishment over the Control group (Figure 4D–F).

Additional upregulation of DEGs related to epidermal differentiation was observed in the RAS+EGF group compared to the RAS group alone (Figure S2A,B, Supporting Information). GO enrichment analysis of this group showed increased activity in epithelial cell differentiation and development (Figure S2C, Supporting Information). Immunohistochemical staining was conducted to corroborate the RNA‐seq results. K6, a key marker in keratinocyte activation postinjury, and K14, indicative of proliferative basal cells in the epidermis, were examined. On day 7 post‐treatment, the experimental groups, especially RAS+EGF and RAS+EGF+VEGF, showed more pronounced K6 expression than the Control group (Figure 4G,H). Similarly, K14 expression was significantly higher in the experimental groups, with the RAS+EGF+VEGF group exhibiting notably elevated K14 levels in the basal layer of the epidermal wound edge (Figure 4I,J). These findings collectively indicate that RAS with dual growth factor gradients facilitates epidermal regeneration.

### 2.5 The Pro‐Angiogenesis Effect of RAS with Dual Growth Factors Gradients in Wound Healing

**Figure 5:**
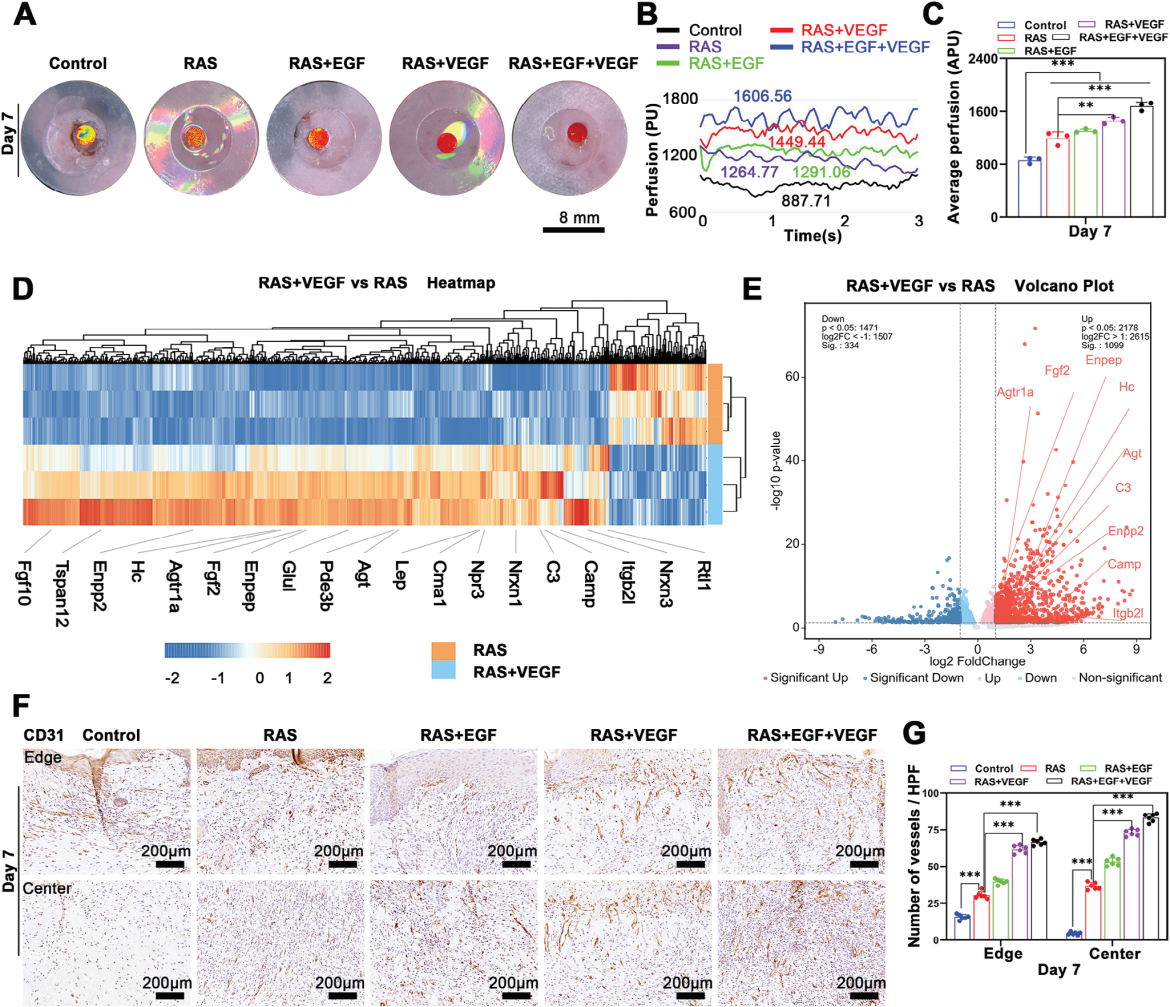
The pro‐angiogenetic effect of RAS with VEGF gradient during wound healing. A) Pseudo‐color maps indicate blood perfusion on the wound area of the Control, RAS, RAS+EGF, RAS+VEGF, and RAS+EGF+VEGF groups after 7 days of treatment. B,C) Dynamic detection and quantification of blood perfusion across the Control, RAS, RAS+EGF, RAS+VEGF, and RAS+EGF+VEGF groups after 7 days of treatment. D,E) The heatmap and volcano plot illustrates the differentially expressed genes related to angiogenesis activities of the RAS+VEGF group compared to the RAS group. F) Immunohistochemical staining of CD31 in wound center area of Control, RAS, RAS+EGF, RAS+VEGF, and RAS+EGF+VEGF groups after 7 days of treatment. G) The quantification of CD31 expression in wound center area of Control, RAS, RAS+EGF, RAS+VEGF, and RAS+EGF+VEGF groups after 7 days of treatment. ^**^ *p* < 0.01,^***^ *p* < 0.001.

Angiogenesis in the wound area was assessed on the 7th day using a Laser speck imaging system, which monitored blood flow. Pseudo‐color maps and blood perfusion analysis of representative samples indicated an incremental increase in blood perfusion volume across the groups: Control, RAS, RAS+EGF, RAS+VEGF, and RAS+EGF+VEGF, with values of 887.71, 1264.77, 1291.06, 1449.44, and 1606.56, respectively (**Figure** **5**A,B). The experimental groups showed a higher average wound perfusion volume than the Control group, with the RAS+EGF+VEGF group exhibiting the most substantial increase (Figure 5C).

RNA‐seq analysis on the 7th day postwounding identified differentially expressed genes (DEGs) between the RAS and RAS+VEGF groups. Heatmaps and Volcano Plot study highlighted numerous DEGs, especially those associated with angiogenesis (Figure 5D,E). Immunohistochemistry was used to evaluate neovascularization in the wound. The results revealed more CD31‐positive cells in the RAS+VEGF and RAS+EGF+VEGF groups than the other three groups. The Control group showed minimal neovascularization (Figure 5F,G).

### 2.6 The Inflammation Regulation Effect of RAS with Dual Growth Factors Gradients

**Figure 6:**
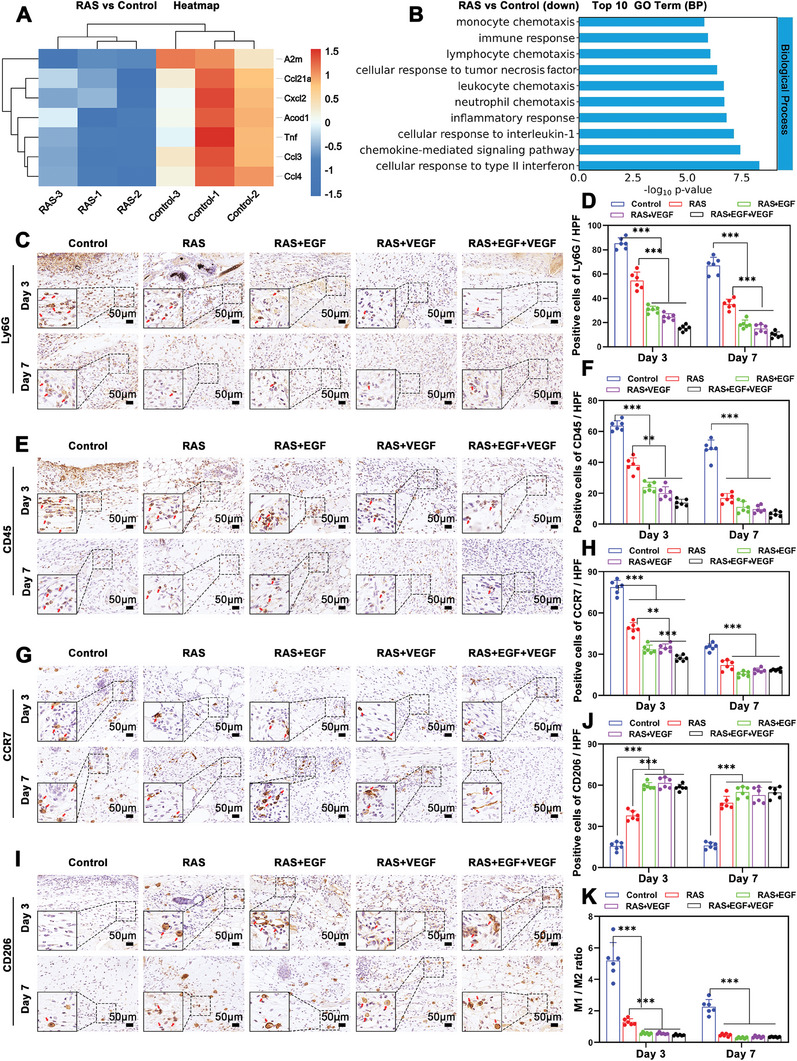
The inflammatory responses of RAS with dual growth factors gradients. A) The heatmap illustrates the differentially expressed genes related to the inflammatory down‐regulation ability of RAS compared to the Control group. B) The GO function enrichment analysis of these differentially expressed genes. C,D) The expression of Ly6G (a pan marker of monocytes, granulocytes, and neutrophils) in the wound center area of the Control, RAS, RAS+EGF, RAS+VEGF, and RAS+EGF+VEGF groups after 3 and 7 days of treatment. E,F) The expression of CD45 (a pan leukocyte marker) in the wound center area of the Control, RAS, RAS+EGF, RAS+VEGF, and RAS+EGF+VEGF groups after 3 and 7 days of treatment. G,H) The expression of CCR7 (M1 macrophage) in the wound center area of the Control, RAS, RAS+EGF, RAS+VEGF, and RAS+EGF+VEGF groups after 3 and 7 days of treatment. I,J) The expression of CD206 (M2 macrophage) in the wound center area of the Control, RAS, RAS+EGF, RAS+VEGF, and RAS+EGF+VEGF groups after 3 and 7 days of treatment. K) The ratio between the numbers of M1‐type macrophages and the numbers of M2‐type macrophages on day 3 and day 7. ^**^ *p* < 0.01, ^***^ *p* < 0.001.

The RNAseq heatmap and volcano plot revealed that the significantly downregulated genes were primarily implicated in the inflammatory response, including the regulation, activation, differentiation, and chemotaxis of inflammatory cells (**Figure** **6**A; Figure S3A, Supporting Information). GO enrichment analysis showed a significant down‐regulation in the RAS group of GO terms related to biological processes such as inflammatory response, leukocyte and neutrophil chemotaxis, monocyte chemotaxis, and cell reactivity to inflammatory factors (Figure 6B). KEGG pathway analysis indicated a substantial down‐regulation in the RAS group of pathways, including NF‐kappa B signaling pathway, TNF signaling pathway, and IL‐17 signaling pathway (Figure S3B, Supporting Information).

Immunohistochemical staining was used to validate these findings. On the 3rd and 7th day post‐treatment, the expression of Ly6G, a marker for monocytes, granulocytes, and neutrophils, was significantly reduced in the wound area for the RAS and growth factor‐treated groups compared to the Control group, with the most substantial reduction in the RAS+EGF+VEGF group (Figure 6C,D). CD45 expression, a pan leukocyte marker, was also lower in the experimental groups compared to the Control group, with even lower levels in the growth factor‐treated groups than in the RAS group alone (Figure 6E,F). Furthermore, CD68 expression, indicative of total macrophages, was decreased in both RAS and growth factor‐treated groups compared to the Control group (Figure S4A,B, Supporting Information). Notably, the expression of CCR7, a marker for M1 macrophages, was reduced in the wound area of the experimental groups compared to the Control group (Figure 6G,H). Conversely, CD206 expression, a marker for M2 macrophages, was significantly higher in the experimental groups, especially in the growth factor‐treated groups (Figure 6I,J). By the seventh day post‐treatment, the M1/M2 macrophage ratio was significantly below one in the experimental groups, suggesting that RAS and growth factors may facilitate a shift in wound inflammation from a pro‐inflammatory to a pro‐repair state (Figure 6K).

### 2.7 The Healing Quality Assessment After Skin Wound Healing

**Figure 7:**
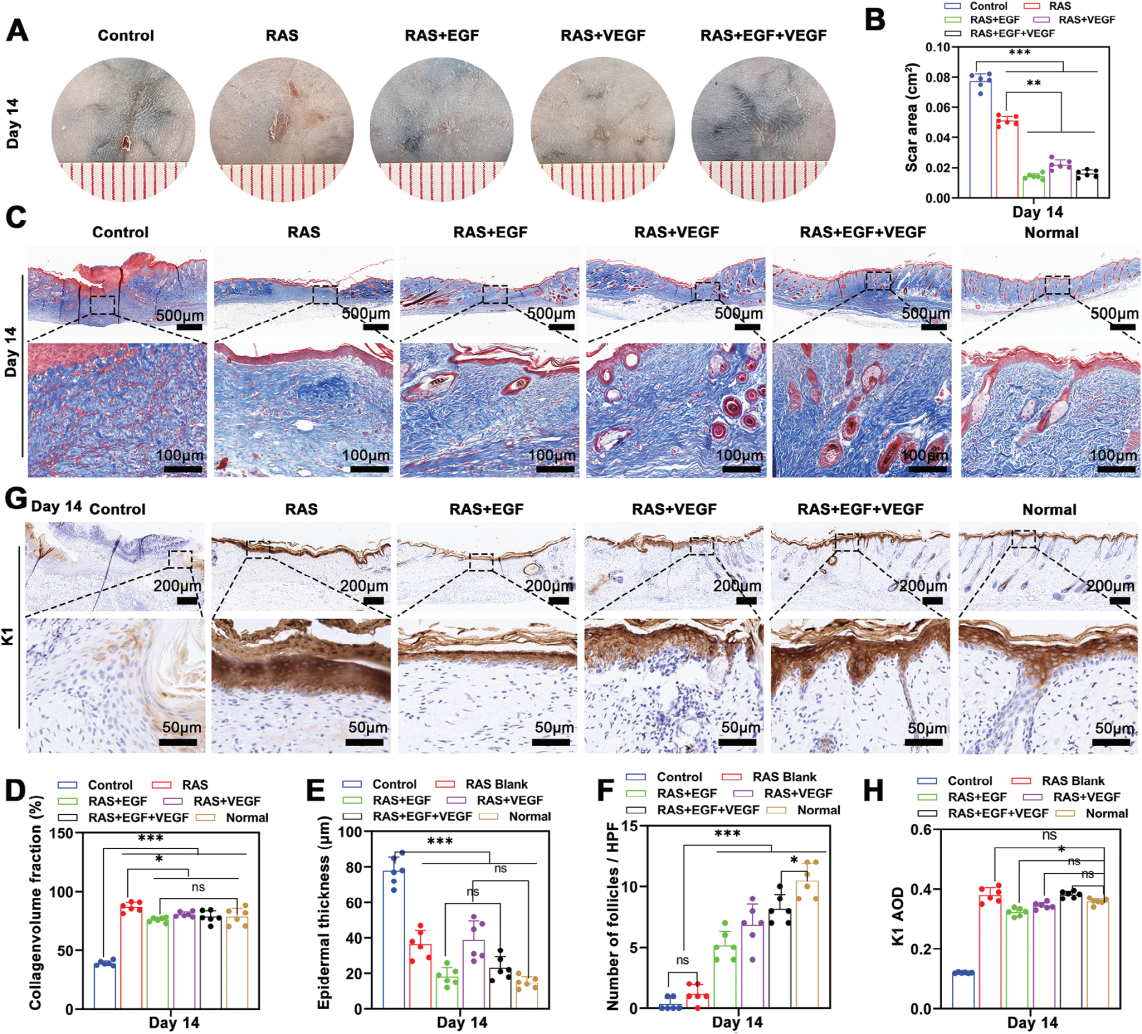
The healing quality assessment after wound closure. A) The photographs of the wound area of the Control, RAS, RAS+EGF, RAS+VEGF, and RAS+EGF+VEGF groups after 14 days of treatment. B) The scar area of the Control, RAS, RAS+EGF, RAS+VEGF, and RAS+EGF+VEGF groups after 14 days of treatment. C) The trichrome staining of the wound area of the Control, RAS, RAS+EGF, RAS+VEGF, and RAS+EGF+VEGF groups after 14 days of treatment. D) The evaluation of collagen volume fraction of the Control, RAS, RAS+EGF, RAS+VEGF, and RAS+EGF+VEGF groups after 14 days of treatment. E,F) The epidermal thickness and regenerated follicles in the wound area of the Control, RAS, RAS+EGF, RAS+VEGF, and RAS+EGF+VEGF groups after 14 days of treatment. G,H) The expression of K1 in the wound center area of the Control, RAS, RAS+EGF, RAS+VEGF, and RAS+EGF+VEGF groups after 14 days of treatment. ^*^ *p* < 0.05, ^**^ *p* < 0.01,^***^ *p* < 0.001.

We evaluated the impact of RAS with dual growth factor gradients on healing quality that focuses on scar area, ECM remodeling, and epidermal maturation. On day 14, representative images showed complete wound closure in the experimental groups, while the control group still had residual scabs. The RAS+EGF+VEGF group had the smallest scar area (**Figure** **7**A,B). Masson's trichrome staining on day 14 post‐treatment revealed dense collagen deposition in all groups. However, the control group's granulation tissue had numerous new blood vessels, whereas the RAS, RAS+EGF, RAS+VEGF, and RAS+EGF+VEGF groups showed collagen density and arrangement akin to normal skin (Figure 7C,D).

Epidermal thickness measurements showed that the RAS+EGF and RAS+EGF+VEGF groups had thicknesses comparable to normal epidermis (Figure 7E). Skin appendage regeneration, contributing to reduced scarring, was evident on day 14 postwounding. Hair follicle regeneration was noticeable in the RAS+EGF, RAS+VEGF, and RAS+EGF+VEGF groups. Despite fewer hair follicles than normal skin, these groups still exceeded the control and RAS groups, suggesting that growth factor supplementation can enhance wound healing quality (Figure 7F). K1, a differentiation‐specific protein that increases during keratinocyte terminal differentiation, indicates epidermal maturation. Immunohistochemical analysis showed significantly higher K1 expression in the experimental groups compared to the control group, nearing levels in normal skin, which signifies mature‐stage epidermal regeneration in the experimental groups (Figure 7G,H).

## 3 Discussion

The healing of skin wounds is a complex process requiring the orchestrated interplay of re‐epithelialization, angiogenesis, and immune response modulation. Our study introduces a radially aligned scaffold (RAS) with dual growth factor gradients designed to enhance these processes for improved wound healing and functional skin reconstruction. The RAS's radial structure is not merely a physical framework but a functional guide for cell migration, promoting rapid and directed cell adhesion and pseudopodia extension. Integrating EGF and VEGF into the scaffold provides a localized stimulus for epidermal regeneration and vascularization, leading to more efficient coverage of the wound area and increased blood supply.

The innovation of our approach lies in the dual gradient of growth factors within the 3D radially aligned spatial structure of the nanofiber scaffold. This design is inspired by the natural biological gradients in tissues,^[^ ^15^ ^]^ which provide cues for cell orientation, migration, and function. In the presented RAS, a VEGF gradient decreases from the center to the periphery, enhancing the angiogenetic, as it ensures an adequate blood supply to the wound bed,^[^ ^17^ ^]^ essential for delivering nutrients and oxygen necessary for tissue repair.^[^ ^16^ ^]^ The VEGF gradient within the RAS was designed to attract and stimulate the proliferation of vascular endothelial cells,^[^ ^17^ ^]^ particularly in the central area of the wound, which is often the most challenging region to vascularize. By the seventh day post‐treatment, the VEGF‐treated wounds exhibited a marked increase in blood perfusion volume and angiogenic gene expression, indicating that the scaffold's structural and compositional gradients synergistically promote angiogenesis.

EGF gradient decreases from the periphery to the center, which aims to recruit and regulate the activity of keratinocytes. First, we found RAS treatment showed a significantly faster healing rate and improved re‐epithelialization. Gene expression analysis further supported these findings, with an upregulation of genes associated with keratinocyte differentiation and keratinization in the RAS‐treated groups. Markers of keratinocyte activity, such as K1 (epidermal maturation),^[^ ^18^ ^]^ K6, and K14 (proliferation of epidermal basal cells),^[^ ^19^ ^]^ were also more pronounced in the RAS group, indicating that the scaffold not only provides a structure for cell growth but also actively promotes cell proliferation and migration. The above results are own to regulatory effects of nano topographic cues of RAS on keratinocytes, which belongs to physical regulation and has been confirmed by many colleagues.^[^ ^10^, ^20^ ^]^ In addition, EGF acts on keratinocytes, triggering their activation and migration toward the wound center.^[^ ^21^ ^]^ By establishing an EGF gradient within the RAS, we aimed to harness this cytokine's ability to regulate the activities of keratinocytes, thereby enhancing the scaffold's capacity to promote epidermal regeneration. Our findings confirm that the EGF gradient within the RAS significantly upregulated genes related to epidermal differentiation, contributing to the scaffold's efficacy in promoting skin regeneration.

Inflammation regulation is another critical aspect of wound healing.^[^ ^22^ ^]^ An excessive or prolonged inflammatory response can impede healing and lead to scar formation. Our RAS modulated the inflammatory response effectively, reducing pro‐inflammatory markers and facilitating a shift toward a pro‐repair state. Incorporating dual growth factors into the RAS further enhanced this regulatory effect, improving healing outcomes. This modulation of the immune microenvironment, along with the promotion of epidermal maturation and hair follicle regeneration, resulted in reduced scarring and improved the overall quality of wound repair.

## 4 Conclusion

In this study, we have developed a radially aligned scaffold (RAS) with dual growth factor gradients that significantly enhances skin wound healing. The scaffold's architecture, featuring aligned nanofibers, promotes efficient cell migration and rapid coverage of the wound area. The incorporation of epidermal growth factor (EGF) fosters the proliferation and differentiation of epidermal cells. In contrast, vascular endothelial growth factor (VEGF) stimulates angiogenesis, which is crucial for supplying nutrients and oxygen to the healing tissue. Our findings demonstrate that the RAS+EGF+VEGF group outperforms other groups in promoting wound closure, reducing scar formation, and facilitating the regeneration of skin appendages. This scaffold system accelerates healing and improves the quality of the repaired tissue, indicating its potential as a superior treatment modality for skin injuries. The success of this scaffold suggests a promising direction for future research in tissue engineering and regenerative medicine, with the potential for significant clinical impact.

## 5 Experimental Section

### Materials and Reagents

Polycaprolactone (MW: 80 kDa), pluronic‐F‐127, and gelatin were ordered from Sigma‐Aldrich (St. Louis, USA). Sodium borohydride (catalog no. S432209) was purchased from Aladdin (Shanghai, China). Penicillin‐streptomycin (catalog no. 15140122), fetal bovine serum (catalog no.10099‐141C), and Dulbecco's modified eagle medium (catalog no. 11965118) were obtained from Gibco (Shanghai, China). FITC‐BSA (catalog no. bs‐0292P‐FITC) was purchased from Bioss (Beijing, China). Cy5‐NH2 (catalog no. 807529‐70‐91) was purchased from CONFLUORE (Xian, China). BeyoCUBIC (catalog no. P0112M) tissue‐clearing reagent was purchased from Beyotime (Shanghai, China). Anti‐CD31 rabbit pAb (catalog no. ab28364), anti‐α‐SMA rabbit mAb (catalog no. ab150301), anti‐K14 rabbit pAb (catalog no. ab7800), anti‐K1 rabbit pAb (catalog no. ab185629), anti‐CD45 rabbit pAb (catalog no. ab10558), anti‐CD68 rabbit pAb (catalog no. ab283654), and anti‐ CCR7 rabbit pAb (catalog no. ab253187) were ordered from Abcam (Cambridge, UK). Anti‐K6 rabbit pAb (catalog no. bsm‐60235R) was ordered from Bioss (Beijing, China). Anti‐CD206 rabbit pAb (catalog no. PA5‐101657) was ordered from Invitrogen (California, USA). Anti‐Ly6G rabbit pAb (catalog no. 551459) was ordered from BD Pharmingen (California, USA)

### Fabrication of RAS

The RAS (radially aligned scaffold) was prepared following an established protocol. Initially, a directional nanofiber mat with a thickness of 1 mm was created by electrospinning a solution of 10% polycaprolactone (PCL) and a 1% solution of Pluronic F‐127. Once the nanofiber mat was prepared, it was sectioned into small squares measuring 1 by 1 cm submerged in liquid nitrogen. Next, the squares were thermally fixed by applying heat in a direction orthogonal to the nanofibers' alignment, but only on one side of the mat. This unilaterally thermally fixed mat was then submerged in a 1 m solution of sodium borohydride. This immersion led to the expansion of the mat, causing it to form a cylindrical shape through a process of rotational expansion. After this transformation, the scaffold was cleaned using distilled water and freeze‐dried. To further enhance each scaffold's mechanical properties, they were soaked in a 1% gelatin solution for 1 h and freeze‐dried. The final step in the fabrication process involved slicing the gelatin‐coated scaffold into 1 mm thick sections. These sections were then shaped into circular pieces with diameters of 8 and 6 mm using a punch biopsy, ensuring uniformity and consistency in the final scaffold dimensions.

### SEM Observation of RAS Morphology

The RAS sample was subjected to Pt sputtering for 1 min (High Vacuum Ion Sputtering Instrument, Leica, EM ACE600), followed by imaging under SEM (Field Emission Scanning Electron Microscopy, Hitachi, SU8010) at a 5 kV acceleration voltage. Subsequently, the nanofiber orientation of the scaffold and the average pore diameter were quantified based on analysis of acquired SEM images.

### In Vitro Cytocompatibility of RAS

The scaffolds were disinfected with ultraviolet radiation for 24 h and subsequently placed in 48‐well plates. The 50 µL suspension of Human Umbilical Vein Endothelial Cells (HUVECs, 1 × 104 labeled with red fluorescent) was inoculated onto each scaffold, allowing the cells to adhere to the surface for 2 h before supplementation with a complete medium. The samples were cultured at 37 °C and under an atmosphere containing 5% carbon dioxide for 1, 3, 7, and 10 days, respectively. The culture medium was ECM supplemented with a concentration of FBS equal to 10%. Three replicates were performed at each indicated time point.

### Confocal Imaging and SEM Observation of HUVECs on RAS

The cell samples cultured to specific time points were extracted and fixed using 4% paraformaldehyde. Subsequently, they underwent three rounds of PBS cleaning and visualized using a confocal laser microscope (HR Confocal Laser Scanning Microscope, Nikon, A1). The Z‐stack imaging covered a range from 0 to 1000 µm, with intervals of 10 µm. The fixed sample was also subjected to a gradient alcohol dehydration process followed by treatment with a critical point dryer (Leica, EM CPD3000) to obtain a wholly dried sample. Subsequently, the dried samples were subjected to sputtering techniques, and the cellular morphology of each sample was examined using scanning electron microscopy at a 5 kV acceleration voltage.

### Preparation and Characterization of RAS with Dual Composition Gradients

*Fluorescent Dye Simulated Drug Loading*. The drug loading was simulated using fluorescent dyes to establish a spatial distribution. A solution of 8 uL 0.3% FITC was precisely dispensed at the central region of the 8‐mm scaffolds, generating a gradient from the center toward the periphery. Subsequently, uniform loading of 4 uL 0.5% Cy‐5 solution was achieved around the 8‐mm scaffolds, resulting in a gradient extending from the edge toward the center. Confocal imaging effectively visualized and characterized these distinct gradients formed by two fluorescent dyes. Quantitative analysis based on confocal images enabled the determination of fluorescence intensity for both dyes within and surrounding the scaffold.

*Preparation of Different RAS for Subcutaneous Implanting*. A 4 µL solution of FGF at a concentration of 0.05 µg µL^−1^ (containing 200 ng FGF) was applied to the center of a 6‐mm diameter RAS to create a center‐to‐periphery FGF gradient. Subsequently, a uniform loading of 2 µL VEGF solution at a concentration of 0.1 µg µL^−1^ (containing 200 ng VEGF) was applied ≈6‐mm diameter RAS to form a peripheral‐to‐center VEGF gradient.

*Preparation of Different RAS for the Wound*. The RAS center with a diameter of 8 mm was subjected to adding 8 µL VEGF solution (0.025 µg µL^−1^, containing 200 ng VEGF), establishing a center‐to‐periphery VEGF gradient. Subsequently, a uniform loading of 4 µL EGF solution (0.05 µg µL^−1^, containing 200 ng EGF) was performed ≈8‐mm scaffold to generate an EGF gradient from the periphery toward the center.

### Subcutaneous Implantation

The Animal Care Committee at the Wenzhou Institute, University of Chinese Academy of Sciences in Wenzhou, China, approved the animal testing procedures by regulations. Animal Experimental Ethics Number: WIUCAS22120901 and WIUCAS23021606. All animals were obtained from the Zhejiang Experimental Animal Center. Subcutaneous implantation experiments were conducted to assess the biosafety of the scaffold in vivo and validate drug gradients. Following anesthesia with a mixture of 2% isoflurane and 1% oxygen, mice were prepared by shaving hair and disinfecting their back with iodophor. Incisions measuring 6 mm wide were made on both sides of their bare backs. The animals were randomly divided into RAS, RAS+FGF, RAS+VEGF, and RAS+FGF+VEGF. Subsequently, the prepared 6‐mm scaffolds of each group were subcutaneously implanted at each incision site, and wound closure was achieved using 6‐0 nylon thread for suturing purposes. A sterile dressing (Tegaderm, 3 m, St. Paul, MN) was applied to cover the closed wound. Scaffolds were collected after seven days for subsequent histological evaluation.

### Tissue Clearing

The mice's blood vessels were effectively emptied by perfusion with a 4% paraformaldehyde solution, and the resulting samples were fixed in this solution for another 24 h. After fixation, the samples were washed thrice with 1x Wash Buffer for 2 h each time and then immersed in a 50% BeyoCUBIC‐I Solution for incubation continued for 24 h at 37 °C on a shaker set at 60 rpm. Then, the liquid was replaced with 100% Solution I for another 24 h. This immersion process was repeated daily until the tissue sample achieved complete transparency. Following three additional washes with 1X Wash Buffer, the sample was incubated with GS‐IB4 antibody (1:500) for three days (away from light). Subsequently, six rounds of washing with 1X Wash Buffer were performed at room temperature, with each cycle enduring for 2 h. After immersing the sample in BeyoCUBIC‐II Solution (50%), it was incubated at 37 °C on a shaker set at 60 rpm for 24 h until it settled to the tube's bottom. Then, the liquid was replaced with 100% Solution II for another 24 h. After entirely removing Solution II, Mounting Solution was added to immerse the sample for 10 min before placing it onto confocal petri dishes for photography.

### Wound Healing Assessment in Vivo

Male C57 mice aged 8–12 weeks were anesthetized with a mixture of 2% isoflurane and 1% oxygen and placed on a heated pad to maintain body temperature. The dorsal area measuring 4 × 4 cm^2^ was shaved, followed by three applications of the povidone‐iodine solution to the exposed skin, which was subsequently wiped with an alcohol cotton pad three times. Later, full‐thickness excision wounds with an 8 mm diameter were created bilaterally on the back of each mouse using a hole punch (with a gap between the two wounds measuring 1.5–2 cm). Silicone fixation rings were used to secure the wounds for an antishrinkage model to prevent contraction. After establishing a full‐thickness skin injury model, mice were randomly assigned to one of five groups: Control group, RAS group, EGF group, VEGF group, and EGF+VEGF group (*n* = 3 per group). After the implantation of these scaffolds in each group, the wounds were covered with a sterile dressing. The wounds were observed daily, as well as photographed at 3, 7, 10, and 14 days. Tissue samples were collected at these specific time points. The calculation of wound closure rate: Wound closure rate (%) = (Si – St) / Si x 100%. Si represented the initial wound area, and St represented the wound area at 3, 7, 10, and 14 days.

### Microcirculation Blood Perfusion and Distribution Testing in Subcutaneously Implanted Tissue and Wound Tissue

For subcutaneous implantation tissue, the mice were subjected to gas anesthesia before carefully opening the wound suture to separate the subcutaneous and muscular layers, ensuring the integrity of the tissues that enclosed the scaffold. The blood perfusion and distribution of tissues were quantified using a Laser speckle imaging system (RWD, RFLSI III‐SE). RFLSI III‐SE was employed to measure the blood flow of wound tissue on the 7th day postinjury.

### RNAseq and Bioinformatics Analysis

TRIzol reagent was utilized for total RNA extraction, and RNA purity assessment, quantification, and transcriptome library construction were carried out. OE Biotech Co., LTD (Shanghai, China) conducted the analysis and sequencing of the transcriptome. The Illumina Novaseq 6000 platform was used to sequence the libraries, producing 150 bp paired‐end reads. DESeq2 software was applied for differential gene expression analysis with a consistent q‐value threshold of ≤0.05 and fold change criteria of >2 or <0.5, defining differentially expressed genes (DEGs). Hierarchical cluster analysis using R (v 3.2.0) was performed to illustrate gene expression patterns across various groups and samples. Subsequently, Using the hypergeometric distribution algorithm, GO and KEGG Pathway enrichment analyses were conducted on the DEGs to identify significantly enriched functional terms. The GSEA software was utilized to conduct gene set enrichment analysis.

### Histological Observations

The wound tissue was collected on the 3rd,7th, 10th, and 14th day postinjury and subjected to histological analysis. The intact wound tissue was fixed using a solution of 4% paraformaldehyde, dehydrated with a gradient of alcohol, clarified with xylene, followed by wax embedding. Subsequently, the wax blocks were sectioned to obtain paraffin sections with a thickness of 5 µm. Sections from each group were selected for subsequent rehydration and subjected to HE staining and Masson's trichrome staining. The calculation of re‐epithelialization rate: (The distance of re‐epithelialization / total width of the initial wound) x 100%

### Immunohistochemical Staining

The paraffin sections from each experimental group were subjected to dewaxing, rehydration, and antigen retrieval, followed by blocking with a 5% goat BSA solution for 1 h at room temperature. Then, the sections of each group were incubated with primary antibodies against CD45 (1:300), Ly6G (1:200), CCR7 (1:300), CD206 (1:300), CD68 (1:300), CD31 (1:300), K1 (1:200), K14 (1:200) and K6 (1:300) overnight at 4 °C. After washed three times with 1x PBS solution, the sections were treated with HRP‐conjugated secondary antibody for 1 h at room temperature. The color reaction was developed using DAB (diaminobenzidine, 1:20). Finally, the sections were scanned and analyzed using a pathological biopsy scanner.

### Statistical Analysis

The statistical results were reported as mean values with standard deviations (SD). The dataset was analyzed utilizing GraphPad Prism 8.0 software. The one‐way analysis of variance (ANOVA) was conducted to assess the differences among groups. The results were deemed statistically significant when the *p*‐value fell below 0.05.

## Conflict of Interest

The authors declare no conflicts of interest.

## Supporting information

Supporting Information

## Data Availability

The data that support the findings of this study are available from the corresponding author upon reasonable request.
