# Correction to “Hierarchically Assembled Nanofiber Scaffolds with Dual Growth Factor Gradients Promote Skin Wound Healing Through Rapid Cell Recruitment”

**DOI:** 10.1002/advs.202501010

**Published:** 2025-02-08

**Authors:** Ruyi Fan, Chuwei Zhang, Fei Li, Bo Li, Alec McCarthy, Yi Zhang, Shixuan Chen, Lin Zhang

This article corrects the following:

Hierarchically Assembled Nanofiber Scaffolds with Dual Growth Factor Gradients Promote Skin Wound Healing Through Rapid Cell Recruitment.

*Adv. Sci*. **2024**, *11*, 2309993.

https://doi.org/10.1002/advs.202309993

In Figure 1H, the figure was incorrect:


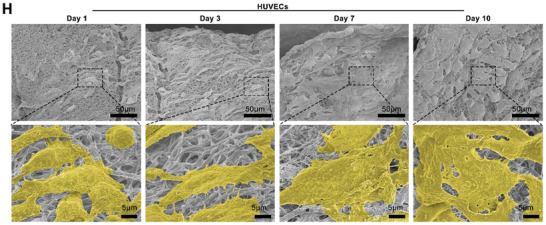


This should be corrected as follows:


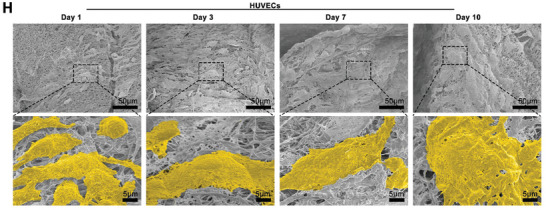


This correction does not affect any conclusions of the paper. We sincerely apologize for any inconvenience caused.

