# Supplementary material for: Hierarchically Assembled Nanofiber Scaffolds with Dual Growth Factor Gradients Promote Skin Wound Healing Through Rapid Cell Recruitment
Source: Adv Sci (Weinh). 2024 Feb 7;11(14):2309993. doi: 10.1002/advs.202309993 (PMC11005683; doi:10.1002/advs.202309993)
Supplement: Supplementary file 1 — Supporting Information [file ADVS-11-2309993-s001.pdf]

ADVANCED SCIENCE
Open Access

Supporting Information

for *Adv. Sci.*, DOI 10.1002/advs.202309993

Hierarchically Assembled Nanofiber Scaffolds with Dual Growth Factor Gradients Promote Skin Wound Healing Through Rapid Cell Recruitment

Ruyi Fan, Chuwei Zhang, Fei Li, Bo Li, Alec McCarthy, Yi Zhang, Shixuan Chen* and Lin Zhang**

Supporting Information

Hierarchically Assembled Nanofiber Scaffolds with Dual Growth Factor Gradients Promote Skin Wound Healing Through Rapid Cell Recruitment

Ruyi Fan, Chuwei Zhang, Fei Li, Bo Li, Alec McCarthy,
Yi Zhang *, Shixuan Chen*, Lin Zhang*

Ruyi Fan, Lin Zhang
Department of Histology and Embryology, School of Basic Medical Sciences, Southern Medical University, Guangzhou 510515, China
National Medical Products Administration (NMPA) and Guangdong Medical Products Administration (GDMPA) Key Laboratory for Safety Evaluation of Cosmetics, Guangzhou 510515, China
Email: zlilyzh@126.com

Chuwei Zhang, Fei Li, Bo Li, Yi Zhang
Department of Burn and Plastic Surgery, Affiliated Hospital of Nantong University, Nantong 226001, China
Email: zhangyi@ntu.edu.cn

Ruyi Fan, Chuwei Zhang, Fei Li, Bo Li, Shixuan Chen
Zhejiang Engineering Research Center for Tissue Repair Materials, Wenzhou Institute, University of Chinese Academy of Sciences, Wenzhou, Zhejiang,325000 China
Email: chensx@ucas.ac.cn

Alec McCarthy
Department of Surgery – Transplant, Holland Regenerative Medicine Program, University of Nebraska Medical Center, Omaha, Nebraska, United States of America

R. Fan and C. Zhang contributed equally to this work.

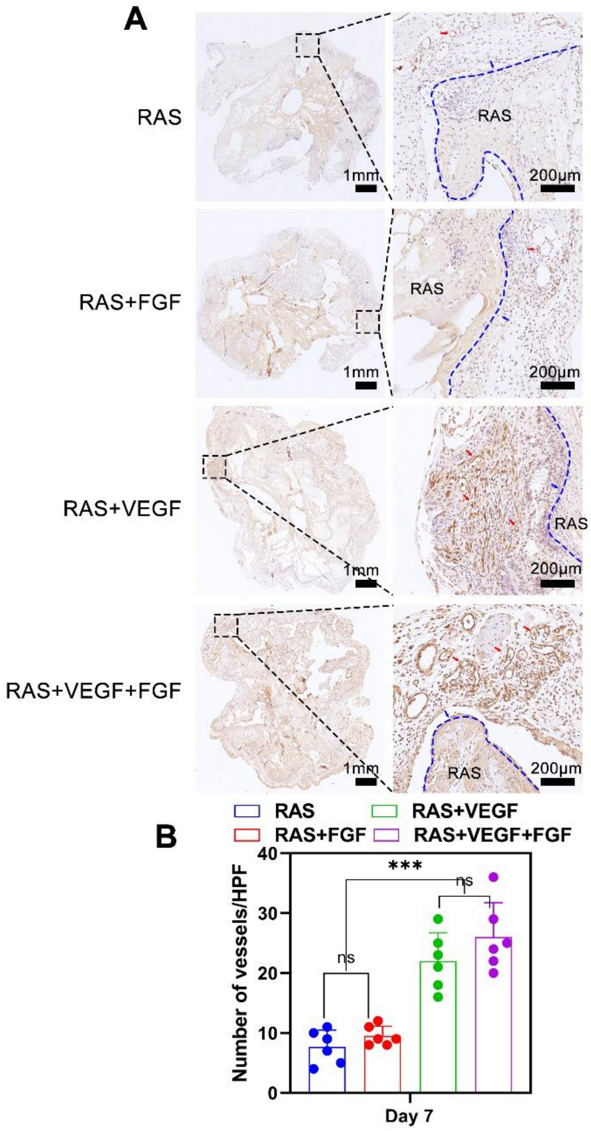

Figure S1. The angiogenetic effect of RAS with dual growth factors. (A) The CD31 expression in the scaffold and surrounding tissue area of the RAS, RAS+FGF, RAS+VEGF, and RAS+VEGF+FGF groups after subcutaneous implantation for one week. (B) The quantification of newly formed blood vessels of the RAS, RAS+FGF, RAS+VEGF, and RAS+VEGF+FGF groups after subcutaneous implantation for one week. ***p<0.001.

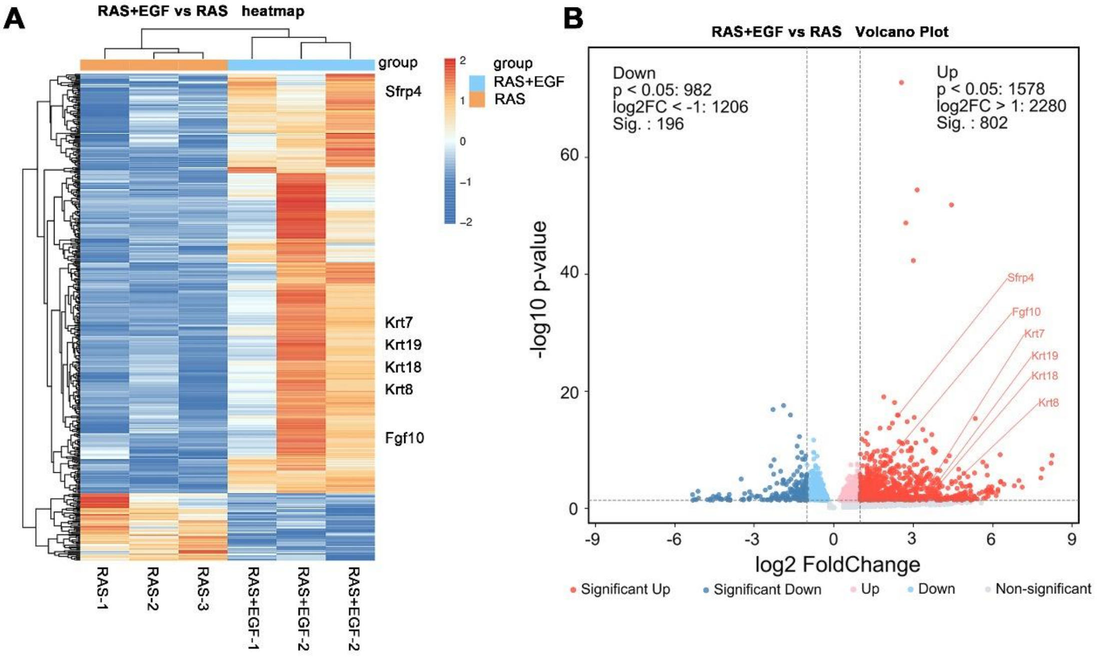

Figure S2. Pro-epidermal regeneration effect of RAS with EGF gradient. (A, B) The heatmap and volcano plot illustrates the differentially expressed genes related to keratinocytes' activities between the RAS+EGF and RAS groups.

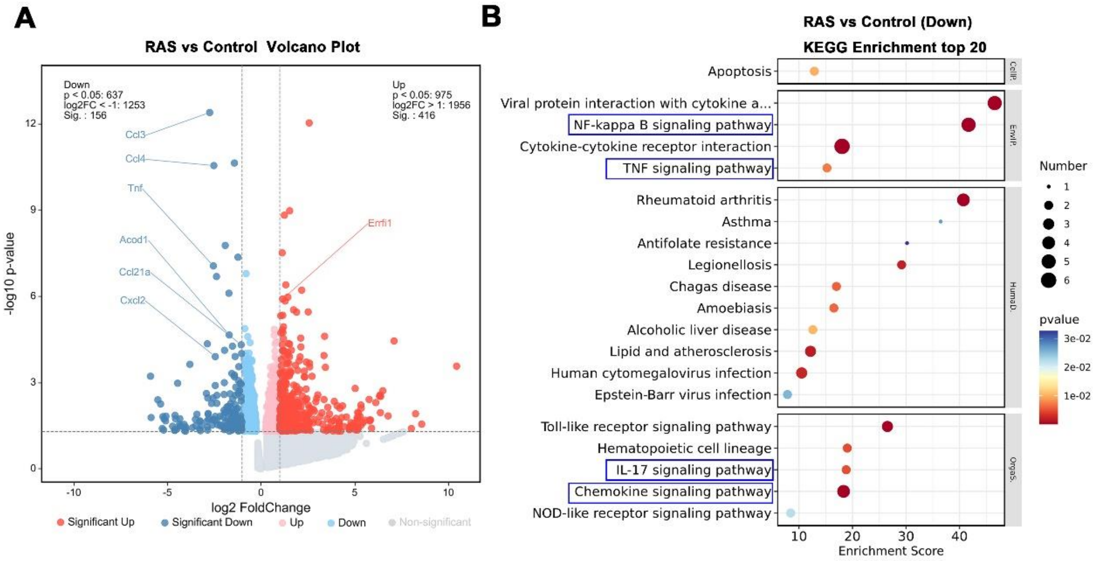

Figure S3. The inflammatory responses of RAS after application. (A) The Volcano Plot illustrates the differentially expressed genes related to the inflammatory down-regulation ability of the RAS compared to the Control group. (B) The Kyoto Encyclopedia of Genes and Genomes (KEGG) enrichment of the RAS group compared to the Control group.

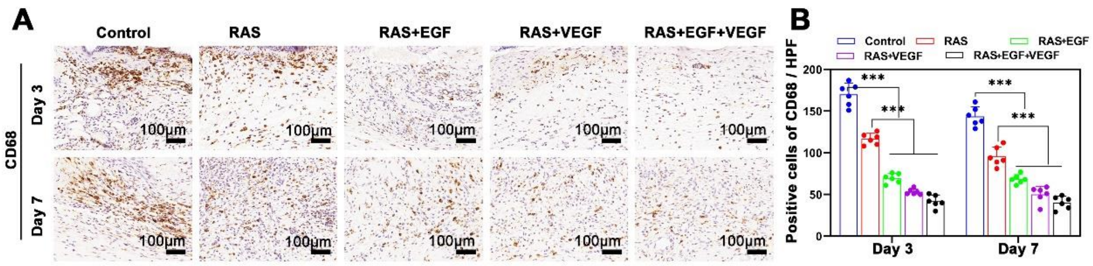

Figure S4. (A, B) The expression of CD68 in the wound center area of the Control, RAS, RAS+EGF, RAS+VEGF, and RAS+EGF+VEGF groups after 3 and 7 days of treatment. ***p<0.001.
